# Synaptotagmin-13 Is a Neuroendocrine Marker in Brain, Intestine and Pancreas

**DOI:** 10.3390/ijms222212526

**Published:** 2021-11-20

**Authors:** Marta Tarquis-Medina, Katharina Scheibner, Ismael González-García, Aimée Bastidas-Ponce, Michael Sterr, Jessica Jaki, Silvia Schirge, Cristina García-Cáceres, Heiko Lickert, Mostafa Bakhti

**Affiliations:** 1 Institute of Diabetes and Regeneration Research, Helmholtz Zentrum München, 85764 Neuherberg, Germany; marta.medina@helmholtz-muenchen.de (M.T.-M.); katharina.scheibner@helmholtz-muenchen.de (K.S.); aimee.bastidas-ponce@helmholtz-muenchen.de (A.B.-P.); michael.sterr@helmholtz-muenchen.de (M.S.); jessica.jaki@helmholtz-muenchen.de (J.J.); silvia.schirge@helmholtz-muenchen.de (S.S.); 2 German Center for Diabetes Research (DZD), 85764 Neuherberg, Germany; ismael.gonzalez@helmholtz-muenchen.de (I.G.-G.); garcia-caceres@helmholtz-muenchen.de (C.G.-C.); 3 School of Medicine, Technische Universität München, 81675 München, Germany; 4 Institute for Diabetes and Obesity, Helmholtz Zentrum München, 85764 Neuherberg, Germany; 5 Medizinische Klinik and Poliklinik IV, Klinikum der Universität, Ludwig-Maximilians-Universität München, 80336 Munich, Germany

**Keywords:** synaptotagmin-13, Syt13, fluorescent reporter, endocrine lineage, brain, intestine, pancreas

## Abstract

Synaptotagmin-13 (Syt13) is an atypical member of the vesicle trafficking synaptotagmin protein family. The expression pattern and the biological function of this Ca^2+^-independent protein are not well resolved. Here, we have generated a novel Syt13-Venus fusion (Syt13-VF) fluorescence reporter allele to track and isolate tissues and cells expressing Syt13 protein. The reporter allele is regulated by endogenous cis-regulatory elements of *Syt13* and the fusion protein follows an identical expression pattern of the endogenous Syt13 protein. The homozygous reporter mice are viable and fertile. We identify the expression of the Syt13-VF reporter in different regions of the brain with high expression in tyrosine hydroxylase (TH)-expressing and oxytocin-producing neuroendocrine cells. Moreover, Syt13-VF is highly restricted to all enteroendocrine cells in the adult intestine that can be traced in live imaging. Finally, Syt13-VF protein is expressed in the pancreatic endocrine lineage, allowing their specific isolation by flow sorting. These findings demonstrate high expression levels of Syt13 in the endocrine lineages in three major organs harboring these secretory cells. Collectively, the Syt13-VF reporter mouse line provides a unique and reliable tool to dissect the spatio-temporal expression pattern of Syt13 and enables isolation of Syt13-expressing cells that will aid in deciphering the molecular functions of this protein in the neuroendocrine system.

## 1. Introduction

Synaptotagmins (SYTs) are membrane trafficking proteins that regulate intracellular vesicle movement and exocytosis. In mammals, this protein family comprises 17 isoforms that are structurally characterized by an extracellular N-terminus region, a transmembrane (TM) domain and two tandem cytoplasmic (C2) domains at the C-terminus [1,2,3]. In several isoforms such as Syt1, Syt2, and Syt7, the C2 domains harbor Ca^2+^-interacting residues and their function requires Ca^2+^-binding [4,5,6,7]. SYTs are mainly expressed in neurons and cell types that possess regulatory secretory pathways. Among these are neuroendocrine cells, which produce and secrete hormones into the blood circulation to regulate different systematic processes such as metabolism [8,9]. The typical Ca^2+^-dependent members such as Syt1 and Syt2 are well-known to mediate synaptic vesicle exocytosis. These proteins bind to the soluble NSF attachment protein receptor (SNARE) proteins and mediate vesicle docking and fusion to the target membranes [2]. Several other SYT isoforms such as Syt4, Syt8, and Syt13 lack the Ca^2+^-binding amino acids and operate in a Ca^2+^-independent manner [7,10]. These Ca^2+^-independent atypical members are less functionally characterized [9].

Syt13 is an atypical SYT protein, which lacks an extracellular N-terminus sequence and is evolutionarily conserved with a high degree of homology between human and rodent sequences [11,12]. Syt13 mRNA is expressed in the brain, heart, lung, testis, spleen, kidney and pancreas [11,12,13]. An increase in the mRNA levels of Syt13 in several brain regions after contextual fear conditioning has been shown [14]. Moreover, Syt13 plays a protective function in motor neurons of patients with amyotrophic lateral sclerosis (ALS) and spinal muscular atrophy (SMA) [15]. Further, SYT13 is upregulated in several cancer cell types, such as gastric and colorectal cancers as well as lung adenocarcinoma. The inhibition of Syt13 using antisense oligonucleotides hampers cancer cell metastasis and progression [16,17,18]. Although these studies have shed some light on the significance of Syt13 in different pathological contexts, the cellular target and functions of this protein are still obscure.

Here, we have generated a novel reporter mouse line by fusing the bright fluorescent protein Venus to the C-terminus of the endogenous Syt13 protein. The resulting homozygous Syt13-Venus fusion (Syt13-VF) mice (Syt13^VF/VF^) were viable and fertile, and Syt13-VF protein pursued the identical expression pattern as the endogenous Syt13 protein. Further, we detected high expression levels of Syt13-VF protein in neuroendocrine lineages in the brain, intestine and pancreas. Finally, the expression of Venus allowed us to specifically isolate Syt13-expressing cells by flow cytometry and track them by live imaging. Overall, the Syt13-VF mouse line offers a unique tool to explore the expression and molecular action of Syt13 in different cell types such as endocrine lineage.

## 2. Results and Discussion

### 2.1. Generation of the Syt13-Venus Fusion Mouse Line

**Figure 1:**
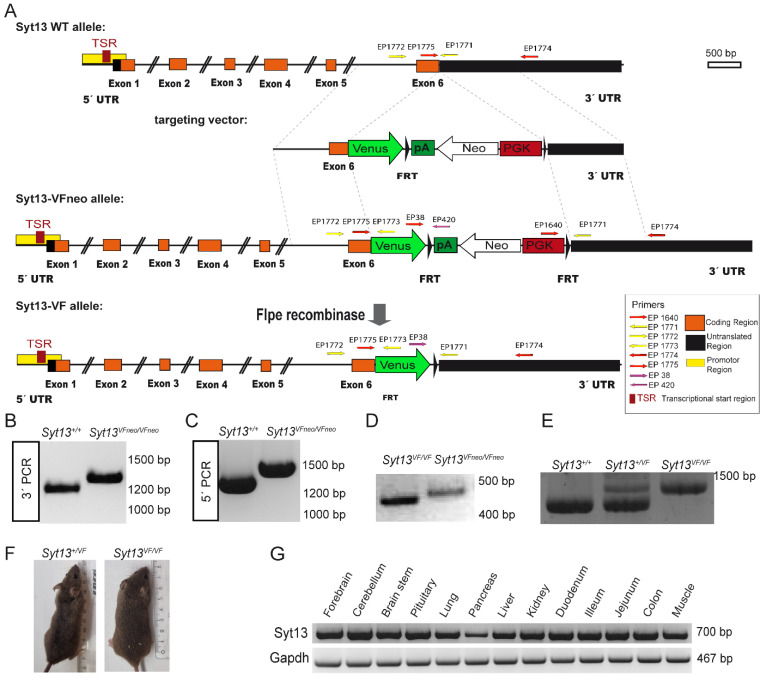
Generation of the Syt13 Venus Fusion (Syt13-VF) allele. (**A**) Targeting strategy of the Syt13-VF allele. A double strand break was performed by two nickases of the D10A mutant Cas9 using two gRNAs binding before and after the stop codon of Syt13. A targeting vector was used to repair the gap and fuse the coding region of the fluorescent reporter gene Venus to the open reading frame. The FRT-flanked PGK-driven neomycin (Neo) selection cassette was removed by Flpe recombinase-mediated excision. Syt13 5′ and 3′ untranslated regions (UTRs) are indicated in black and the predicted promoter region in yellow and the transcriptional start sites (TSR) in red as indicated. Primers used for genotyping EP_1771, EP_1772 and EP_1773 are indicated with yellow arrows. The position of the homology regions to generate the targeting construct are indicated (dashed lines). (**B**,**C**) PCR genotyping of Syt13VFNeo clones using primers EP_1640, EP_1774 and EP1775 (red arrows) confirming the targeted allele Syt13-VFNeo (1335 bp) versus the WT allele (1228 bp) and for 3′PCR and EP_1771, EP_1772, and EP_1773 for 5′PCR (yellow arrows) confirmation of Syt13-VFNeo (1475 bp) versus WT allele (1322 bp). (**D**) PCR primers EP038, EP420, and EP 1771 were used to distinguish the allele before (Syt13-VFNeo; 477 bp) and after removal of the Neo selection cassette (Syt13-VF; 402 bp). (**E**) Primers EP_1771, EP_1772, and EP_1773 were used to distinguish WT from heterozygous or homozygous mice resulting in 1322 bp for the WT allele and 1475 bp for the Syt13-VF allele. (**F**) Side by side image of a WT male mice (left) and a Syt13-VF male mouse (right) of 5 months of age. (**G**) RT-PCR of different tissues of a WT adult mouse presenting bands corresponding to Syt13 and Gapdh transcripts.

To provide a reliable and efficient tool for tracking and isolating cells expressing Syt13 protein, we applied CRISPR/Cas9-mediated double strand breaks and homologous recombination to generate a mouse line, in which Syt13 is fused with the fluorescence protein Venus. We generated a Syt13-Venus fusion (Syt13-VF) reporter allele under control of the endogenous Syt13 cis-regulatory elements (Figure 1A). To do this, we removed the translational stop codon of the Syt13 gene in exon 6 and inserted an in-frame fusion transcript of the Venus open-reading frame. Additionally, we used an FRT-flanked phospho-glycerate kinase (PGK) promoter-driven neomycin (neo) resistance gene as the selection marker. The targeting vector, Cas9D10A expression vector, and two guide RNA vectors expressing guide RNAs (Supplementary Table S1) that bind shortly before and after the Syt13 stop codon were electroporated into IDG3.2 embryonic stem cells (ESCs) [19]. Neomycin resistant clones were screened with 5′ and 3′ homology arm spanning PCRs (Figure 1B,C). Germline chimeras of the Syt13-VFneo mouse line were generated from the aggregation of Syt13-VFneo mESC clone with CD1 morulae. The FRT-flanked neo selection cassette was deleted in the germline by Flpe recombination-mediated excision [20] (Figure 1D), resulting in generation of the Syt13-VF mouse line. The intercross of heterozygous animals (Syt13^+/VF^) produced wild-type (WT, Syt13^+/+^), heterozygous and homozygous (Syt13^VF/VF^) offspring that were genotyped by PCR analysis (Figure 1E). Syt13^VF/VF^ offspring were viable and fertile and appeared indistinguishable from their WT or heterozygous adult littermates (Figure 1F). A 17-month close observation showed no postnatal death, as well as normal sociability, behavior, and health of the reporter mice (132 total animals). The average weight of 6-month-old male mice was 31 g for WT and 32 g for homozygous reporter animals (*n =* 8). The average number of animals per litter in heterozygous intercrosses was 5.75 (*n =* 8), and for homozygous intercrosses it was 5.52 (*n* = 23). Together, these data indicate the successful generation of Syt13-VF allele and the reporter mouse line. To disclose the target tissues for the expression pattern analysis of Syt13-VF protein, we next performed reverse transcription PCR (RT-PCR) of different organ samples isolated from the adult WT mice to identify the expression pattern of Syt13 mRNA. Syt13 was expressed in the brain (forebrain, cerebellum, brainstem, and pituitary), lung, pancreas, liver, kidney, intestine (duodenum, ileum, jejunum, and colon) and muscle (Figure 1G), confirming the broad expression of this gene as has been shown previously [11,12,13]. However, deeper analyses need to be performed in future studies so that the expression pattern of Syt13 can be demonstrated systematically in different organs and tissues. Due to the expression of Syt13 in different areas of brain and intestine, and the prominent expression of SYT members in neuroendocrine cells, we focused on the expression pattern analysis of the Syt13-VF protein in brain, intestine, and pancreas.

### 2.2. Syt13 Is Highly Expressed in Neuroendocrine Cells

**Figure 2:**
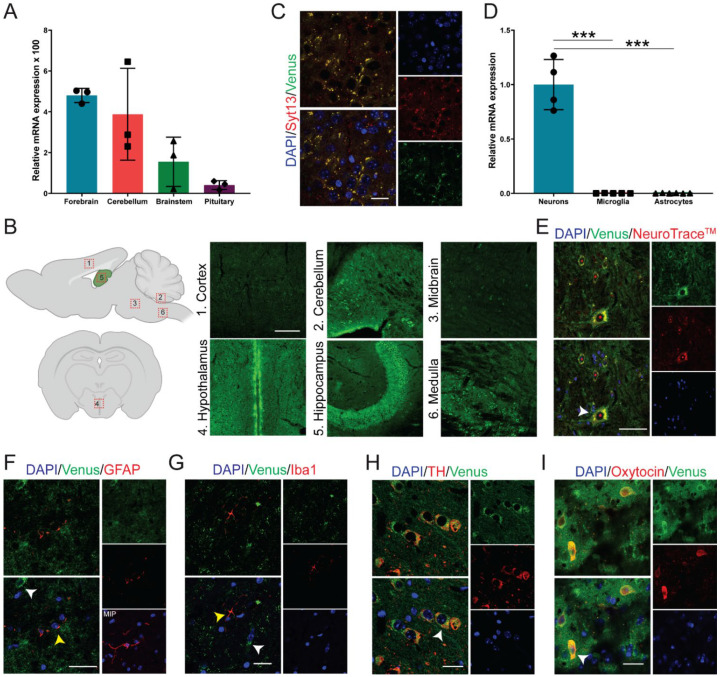
Syt13-VF expression in neuronal tissue. (**A**) Syt13 mRNA expression in different brain regions of adult WT male mice (*n =* 3). Transcript levels were calculated according to the 2^−ΔCt^ method; Gapdh was used as endogenous control to normalize mRNA amount. Bars represent means 2^−ΔCt^ × 100 ± SD. (**B**) Scheme of brain area used for IHC, and maximum intensity projection images of Syt13-VF positive cells at different brain regions including cortex, between IV-VI cortex layers; cerebellum, an area in the proximity of the interposed cerebellar nucleus (anterior part to posterior part) and the medial (fastigial) cerebellar nucleus; midbrain, an area in the proximity of the paramedian raphe nucleus and the pontine reticular nucleus; hypothalamus (coronal orientation), anterior hypothalamus in the 3V proximity; hippocampus, CA2 region area; medulla (sagittal orientation), inferior olive area. Scale bar 100 µm. Scheme was created with BioRender.com (accessed on May 2021). (**C**) Co-staining of GFP and Syt13 in the brain cortex shows co-localization of both markers. Scale bar 20 µm. (**D**) Analysis of Syt13 mRNA expression in neuron and glia cells isolated from WT animals shows high expression of Syt13 in neurons but not in microglia nor astrocytes (*n* ≥ 4). (**E**–**G**) Immunostaining of brain sections confirm the presence of the Syt13-VF protein in neurons marked by NeuroTraceTM, but not in microglia or astrocytes marked by Iba1 and GFAP, respectively. White arrows indicate Syt13-VF and yellow arrows show glia cells. MIP, maximum intensity projection. Scale bar 20 µm. (**H**,**I**) Immunostaining analysis of hypothalamus regions showing colocalization of Syt13-VF with tyrosine hydroxylase (TH) and oxytocin, markers of neuroendocrine cells (white arrows). Scale bar 20 µm. (*** *p* < 0.001; *t*-test). For (**E**–**I**), single confocal images were used to avoid false colocalization. Data are represented as mean ± SD.

To identify the expression pattern of Syt13 in the brain, we first performed quantitative PCR (qPCR) analysis. We detected Syt13 transcripts with high levels in forebrain and cerebellum, and with lower levels in brainstem and pituitary (Figure 2A). Furthermore, we performed immunohistochemical (IHC) analysis of brain sections from Syt13^VF/VF^ mice. Although it was possible to detect the fluorescent signal in the Venus-expressing cells (Supplementary Figure S1A), we used antibodies against Venus protein to amplify the signal, showing the expression of Syt13-VF protein in the cortex, cerebellum, midbrain, hypothalamus, hippocampus, and medulla (Figure 2B). Although the IHC showed variable levels of Syt13-VF in different brain area, further studies are required to quantitatively validate this differential expression levels of Syt13. These data also support a previous study, which has reported a broad expression of Syt13 in several areas of the brain using in situ hybridization [21]. Importantly, co-staining of brain sections with antibodies against Syt13 and Venus disclosed high overlap between the two proteins (Figure 2C). This result indicates that the fusion protein accurately mirrors the expression of the endogenous Syt13 protein. We next explored the expression pattern of Syt13 in different brain cell types. qPCR analysis revealed the expression of Syt13 mRNA in isolated neurons but not in microglia and astrocytes (Figure 2D). Furthermore, IHC of brain sections from Syt13^VF/VF^ mice also revealed the expression of Syt13-VF protein in neurons (marked with NeuroTraceTM) [22] but not in microglia (marked with ionized calcium binding adaptor molecule 1, Iba1) or in astrocytes (marked with glial fibrillary acidic protein, GFAP) (Figure 2E–G and Supplementary Figure S1B). As most SYT members are expressed in neuroendocrine cells, we thus co-stained Venus and neuroendocrine specific markers. We found colocalization of Venus with tyrosine hydroxylase (TH)-expressing and oxytocin-producing cells (Figure 2H,I). These data indicate the high expression of Syt13 in neuroendocrine lineage as it has been reported for several other SYT proteins. However, the precise expression pattern of Syt13 protein in other neuronal cell types need to be further demonstrated in future studies.

### 2.3. Syt13 Expression Is Restricted to Enteroendocrine Cells in the Adult Intestine

**Figure 3:**
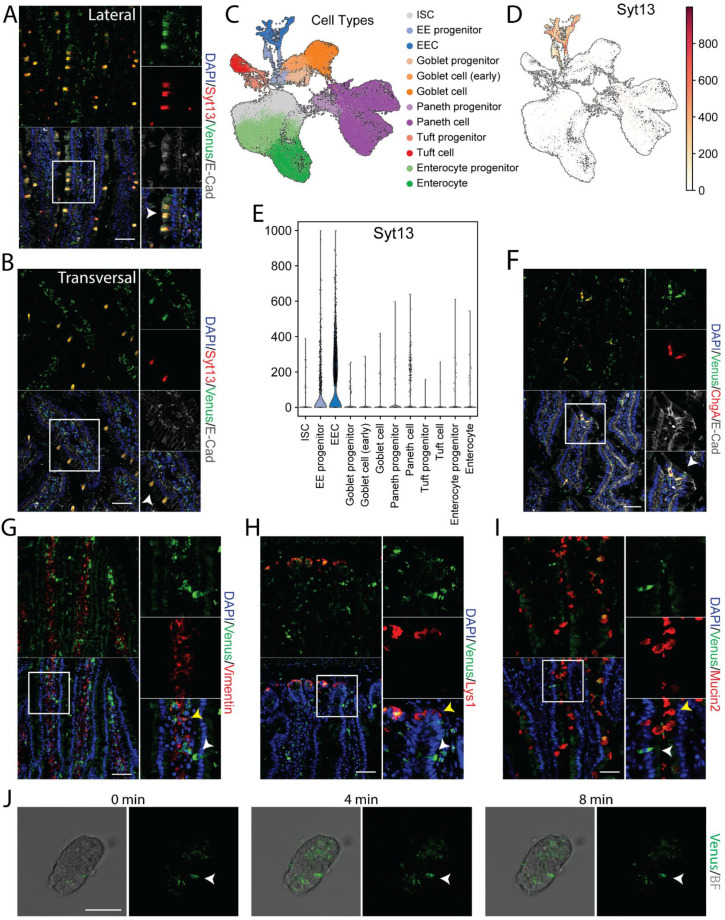
Syt13-VF expression in the adult intestinal epithelium. (**A**,**B**) Co-staining of GFP and Syt13 in intestinal villi from duodenum prepared from the reporter mice. Both markers show co-localization (white arrows) in the lateral and transversal cut. Scale bar 20 µm. (**C**) UMAP plot of 56240 profiled single cells from control mice. Colors highlight clustering into the main cell types and their progenitors. (**D**) Syt13 expression and distribution in UMAP plot. Normalized expression values are shown. (**E**) Violin plot of normalized expression of Syt13 grouped by cell type, showing the highest expression values in enteroendocrine progenitor cells (EE progenitor) and mature enteroendocrine cells (EEC). (**F**) Immunostaining analysis of Syt13-VF and chromogranin A (ChgA) shows colocalization (white arrows) of both markers. Scale bar 20 µm. (**G**–**I**) Syt13-VF does not colocalized with markers for paneth cells, goblet cells, and mesenchymal cells. White arrows indicate Syt13-VF and yellow arrows show cell types. Scale bar 20 µm. (**J**) Time-lapse imaging of an isolated crypt from Syt13-VF reporter mice. White arrows indicate Syt13-VF-expressing cell. Scale bar 50 µm. BF, bright field.

RT-PCR data indicated the expression of Syt13 mRNA in several intestinal areas (Figure 1G). To support these data and identify the expression of Syt13 protein in this organ, we stained intestinal sections from the reporter mice with antibodies against Syt13 and Venus. We detected highly overlapping signals for both antibodies (Figure 3A,B and Supplementary Figure S1C) that not only indicates the expression of Syt13 proteins in the intestine but further confirms the identical expression pattern of the Syt13-VF proteins and endogenous Syt13 in this organ. To dissect in which intestinal cell types Syt13 is mainly expressed, we first reanalyzed the single-cell RNA sequencing (scRNA-seq) data derived from mouse intestinal crypts [23]. Data analysis indicated the expression of Syt13 in all enteroendocrine cells (EECs) and a major fraction of their progenitors but no other intestinal cell types (Figure 3C–E). To confirm these data, we stained intestinal sections from Syt13^VF/VF^ mice for Venus and markers for different intestinal cell types. We found the colocalization of Venus with the EEC marker, Chromogranin A (ChgA) (Figure 3F). However, no colocalization was found with lysozyme 1 (Lys1) (Paneth cells), Mucin 2 (Muc2) (Goblet cells), and Vimentin (Mesenchyme) (Figure 3G–I), demonstrating the restricted expression of Syt13 to the EEC and their progenitors in the intestine. Finally, we executed live imaging of isolated crypts derived from Syt13^VF/VF^ mice. Due to the sufficient fluorescent intensity of the Syt13-VF reporter, we were able to track Syt13 expressing-cells during time-lapse imaging (Figure 3J).

### 2.4. Pancreatic Endocrine but Not Exocrine Cells Specifically Express Syt13 Protein

**Figure 4:**
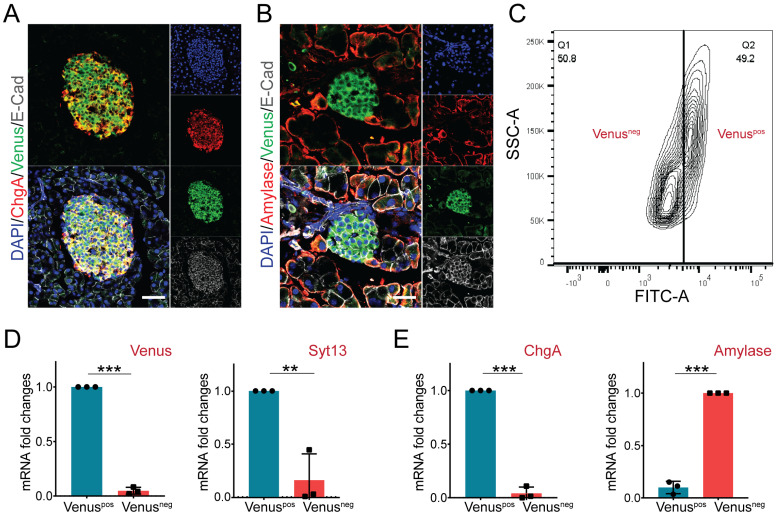
Syt13-VF expression in the adult pancreatic cells. (**A**) Immunostaining analysis of Syt13-VF expression in ChgA-expressing islet cells in pancreatic sections prepared from the reporter mice. Scale bar 20 µm. (**B**) No colocalization between Syt13-VF and amylase-expressing cells is detected. Scale bar 20 µm. (**C**) Representative FACS plot indicating the successful separation of Venus-positive (Venus^pos^) cells from Venus-negative (Venus^neg^) cells. (**D**) qPCR analysis shows high expression levels of Venus and Syt13 in Venus^pos^ cells isolated by FACS (*n* = 3). (**E**) qPCR analysis indicated high expression levels of ChgA and amylase in Venus^pos^ and Venus^neg^ cells, respectively (*n* = 3). (** *p* < 0.01;*** *p* < 0.001; *t*-test). Data are represented as mean ± SD.

Several SYT proteins such as Syt4 and Syt7 are expressed in pancreatic endocrine cells [24,25]. Moreover, islets of Langerhans from patients with type 2 diabetes contain decreased expression levels of Syt13 mRNA [26]. Therefore, we next assessed the expression pattern of Syt13-VF protein in the adult pancreas. Staining of pancreatic sections from Syt13^VF/VF^ mice identified co-expression of Venus with the endocrine lineage marker, ChgA (Figure 4A). Yet, no specific signal for Venus was detected in amylase-expressing acinar cells (Figure 4B). These data suggest restricted expression of Syt13 to the pancreatic endocrine cells. Next, we performed fluorescence-activated cell sorting (FACS) to specifically isolate Syt13-VF-expressing cells. To this end, we performed FACS sorting on a mixture of islets and exocrine tissues (acinar and ductal cells) isolated from the adult pancreas from Syt13^VF/VF^ mice. The bright fluorescence of Venus was sufficient for the successful segregation and isolation of Syt13-VF-positive (pos) and Syt13-VF-negative (neg) cell populations (Figure 4C). We then performed qPCR analysis of the harvested cells and confirmed the expression of both Syt13 and Venus in the Syt13-VF^pos^ population, indicating the capability of the fusion reporter protein for specific isolation of Syt13-expressing cells (Figure 4D). Further, we identified the high expression levels of ChgA and amylase in the Syt13-VF^pos^ and Syt13-VF^neg^ cells, respectively (Figure 4E), indicating the specific expression of Syt13-VF in the pancreatic endocrine lineage.

Altogether, we have generated the first Syt13-Venus fusion reporter mouse line that closely mirrors the expression pattern of the endogenous Syt13 protein. This novel unique tool enabled us to identify the expression of Syt13 at protein level in the endocrine lineage in important organs that regulate systemic metabolism. As the cellular and molecular functions of Syt13 are still unresolved, this new tool will aid in unraveling the function of the protein in (neuro)endocrine cells and neurological disorders. For instance, the fluorescence properties of the Syt13 fusion protein might facilitate the studying of Syt13 functions in the brain by live imaging or electrophysiology. Further, the discovery of the association of Syt13 with different types of cancer also makes this mouse line valuable for the study of Syt13’s role in cancer initiation and progression. As the Syt13-VF mRNA utilizes the Syt13 UTR, it might be possible to use the fusion protein as a sensor to study Syt13 miRNA or antisense oligonucleotide function in silencing Syt13. This is important when aiming to target Syt13 for therapeutic purposes as it has been previously reported [27]. Finally, this novel tool will aid in monitoring Syt13-expressing cells under physiological and pathological conditions in vivo.

## 3. Materials and Methods

### 3.1. Generation of the Targeting Vector

To generate the *Syt13-VF* targeting vector, C57B16 BAC (RPCIB-731L18311Q) was used as the template and the 5′ homology region (HR) and 3′ HR were PCR amplified utilizing the following primers: Fwd primer Ex6A, Rev primer Ex6A (Supplementary Table S1) for 5′ HR and Fwd primer Syt13 Ex6B, Rev primer Syt13 Ex6B for 3′ HR. Then we replaced the 5′ HR via NotI/XbaI and 3′ HR via HindIII/XhoI, from the *Ngn3-VF* targeting vector (pBKS-Venus-RGS-His) to generate the pBKS-*Syt13*-Ex6-Venus construct. Next, by digestion of the PL451-loxP using BamHI and HindII, we obtained the PGK promoter-driven *neomycin* resistance gene flanked by *FRT* sites (FRT-Neo-FRT) and inserted it into the downstream sites of the Venus gene that result in producing the targeting vector pBKS-*Syt13*-Ex6-HR-Venus3xRGS-His-Neo. Using online CRISPR resources, we then designed and generated two gRNA sequences that target up- and downstream near the stop codon of *Syt13* (Figure 1A). Finally, we cloned self-annealed oligos (Syt13 Crispr #1 and #10 fwd and rev; Supplementary Table S1) duplexes with BbsI overhangs into BbsI-digested pBS-U6-chimericRNA (a generous gift from O. Ortiz, Institute of Developmental Genetics, Helmholtz Zentrum München (HMGU)) to generate CRISPR expression vectors. This resulted in the successful generation of pBS-U6-chimericRNA Syt13 #1 and #10 that was confirmed by sequencing.

### 3.2. Embryonic Stem Cell (ESC) Homologous Recombination and Generation of Mouse Line

Mouse ESCs were cultured on a layer of murine embryonic feeder (MEF) layer in Dulbecco’s Modified Eagle Medium (DMEM, Invitrogen, Carlsbad, CA, USA) supplemented with 15% fetal calf serum (FCS, PAN, Aidenbach, Germany), non-essential amino acids (Invitrogen, Carlsbad, CA, USA, 1003), 2 mM L-glutamine (Invitrogen, Carlsbad, CA, USA), 100 μM beta-mercaptoethanol (Invitrogen, Carlsbad, CA, USA), and 1500 U/mL leukaemia inhibitory factor (LIF, Millipore, Darmstadt, Germany, 107 U/mL). We split the cells every 2 days utilizing trypsin (0.05% trypsin, 0.53 mM EDTA; Life Technologies, Darmstadt, Germany). Next, we electroporated a mixture of both pBS-U6-chimericRNA Syt13 #1 and #10, pBKS-*Syt13* Ex6-HR-Venus3xRGS-His-Neo targeting vector and Cas9 nickase overexpression vector (pCAG Cas9v2D10A-bpA; a generous gift from O. Ortiz) into IDG 3.2 mESCs19. We then aggregated the selected clones with CD1 morulae to generate chimeras, which gave germline transmission of the *Syt13-VFNeo* allele. Intercrossing with the *ROSA26:FLPe* mouse line resulted in the removal of the FRT flanked Neo selection marker cassette. The generated mice were kept at the central facilities at HMGU under specific-pathogen-free (SPF) conditions, were kept in a room with a light cycle of 12/12 h, temperature of 20–24 °C and humidity of 45–65%. They received sterile filtered water and were fed with standard diet. All animal studies were conducted with adherence to relevant ethical guidelines for the use of animals in research in agreement with German animal welfare legislation with the approved guidelines of the Society of Laboratory Animals (GV-SOLAS) and the Federation of Laboratory Animal Science Associations (FELASA). Mice were sacrificed with cervical dislocation and post mortem examination of organs was not subject to regulatory authorization. The study was carried out in compliance with the ARRIVE guidelines. For material request contact H.L.

### 3.3. Genotyping

Genotyping was performed through PCR analysis of DNA samples collected from the ear clips. For confirming the excision of the Neo cassette, the primers EP038, EP420 and EP 1771 were used that generated a 477 bp product for the *Syt13-VFNeo* allele and a 402 bp product for the *Syt13-VF delta Neo* allele (Figure 1D). To genotype the heterozygous and homozygous *Syt13-VF* animals, we performed the PCR analysis at a 64 °C annealing temperature using the primers EP 1771, 1772 and 1773. As expected, we found a 1320 bp band for the WT allele and a 1475 bp product for the fusion reporter allele (Figure 1E).

### 3.4. Organ Dissection and Immunostaining

Brain, intestine, and pancreas were dissected and fixed in 4% Paraformaldehyde (PFA) in Phosphate-buffered saline (PBS) overnight at 4 °C. 10% and 30% sucrose solutions were used to cryoprotect the tissues, which were finally incubated in 30% sucrose and tissue embedding medium (Leica, Munich, Germany) (1:1) at 4 °C overnight. Sections of variable sizes were mounted on glass slides (Thermo Fisher Scientific, Darmstadt, Germany) and dried for 10 minutes (min) at room temperature (RT) before use or storage at −20 °C. 1× PBS was applied to the cryosections for rehydration. Afterwards, the samples were permeabilized with 0.2% Triton X-100 in 0.1M Glycine solution for 30 min followed by incubating in a blocking solution (10% FCS, 3% Donkey serum, 0.1% bovine serum albumin (BSA), and 0.1% Tween-20 in PBS) for 1 hour (hr) at RT. Next, they were incubated with the primary antibodies diluted in blocking solution overnight at 4 °C. We used the following primary antibodies for staining: anti-GFP (Chicken 1:1000; Aves Lab; Tigard, OR, U.S.A; GFP-1020), anti-E-Cadherin (Rat 1:300; Kremmer; Dallas, TX, U.S.A; SC-59778), anti-Amylase (Rabbit 1:300; Abcam; Berlin, Germany; AB21156), anti-Syt13 (Rabbit 1:300; Abcam; Berlin, Germany; ab154695), anti-Chromogranin A (Rabbit 1:300; Abcam; Berlin, Germany; ab15160), anti-Lys1 (Rabbit 1:300; Aglient; Santa Clara, CA, U.S.A.; A009902-2), anti-Muc2 (Rabbit 1:300; Santa Cruz; Dallas, TX, U.S.A; sc-15334), anti-Vimentin (Rabbit 1:300; Abcam; Berlin, Germany; ab92547), anti-Oxytocin (Goat 1:1000; Sigma-Aldrich, SAB2501950), anti-TH (Sheep 1:1000; Merck-Millipore; Darmstadt, Germany; AB1542), anti-Iba1 (Rabbit 1:1000; Synaptic Systems, 234 003) and anti-GFAP (Goat 1:1000; Sigma-Aldrich; Munich, Germany; SAB2500462). Samples were then washed with 1x PBS and incubated with the secondary antibodies diluted in blocking solution for 4 hrs at RT. We used secondary antibodies: anti-Rabbit 555 (1:800; Thermo Scientific; Darmstadt, Germany; A31572), anti-Rabbit 647 (1:800; Thermo Scientific; Darmstadt, Germany; A31573), anti-Chicken Cy2 (1:800; Dianova; Hamburg, Germany; 703-225-155), anti-Rat DyLight 549 (1:800; Dianova; Hamburg, Germany; 712-505-153), anti-Goat 555 (1:800; Thermo Scientific; Darmstadt, Germany; A21432) and anti-Mouse 555 (1:800; Thermo Scientific; Darmstadt, Germany; A31570). When needed, DAPI and Neuro TraceTM 640/660 (1:1000, Deep-Red Fluorescent Nissl Stain, Thermo Scientific; Darmstadt, Germany; N21483) diluted in 1x PBS were added for 30 min at RT. Images were captured using a Leica microscope of the type DMI 6000 with the LAS AF software. We analyzed the images utilizing LAS AF (v.4.0; Frankfurt, Germany) and ImageJ (v.1.51 23) software programs (Public Domain).

### 3.5. Neuronal and Glia Cell Isolation

Primary mouse neuronal cultures were obtained from C57BL/6J mouse fetuses at E14. Hypothalami were dissected in ice-cold calcium- and magnesium-free HBSS (Life Technologies, Darmstadt, Germany), digested for 10 min at 37 °C with 0.05% trypsin (Life Technologies; Darmstadt, Germany), washed three times with serum-free MEM supplemented with l-glutamine (2 mM) and glucose (25 mM) and dispersed in the same medium. Cells were cultured in poly-l-lysine (Sigma-Aldrich, St. Louis, MO, USA) coated plates containing MEM supplemented with heat-inactivated 10% horse serum and 10% fetal bovine serum (FBS), 2 mM l-glutamine and glucose (25 mM) without antibiotics. On day 4, half the medium was replaced with fresh culture medium lacking FBS and containing 10 μM of the mitotic inhibitor cytosine-1-β-d-arabinofuranoside (AraC, Sigma-Aldrich; St. Louis, MO, USA) to inhibit non-neuronal cell proliferation and for further experimental analysis.

Primary mouse astrocyte and microglia cultures were obtained from 1-day-old C57BL/6J mouse pups. Hypothalami isolated in ice-cold calcium- and magnesium-free PBS were mechanically dissociated in DMEM-F12 (Gibco, Life Technologies; Darmstadt, Germany) containing 1% antibiotics. The cell suspension was filtered through a 70 μm cell-strainer (BD, Biosciences, Bergen, NJ, USA), centrifuged, and the pellet was resuspended in growth medium (DMEM-F12 supplemented with 10% heat-inactivated FBS and 1% antibiotics) where cells were cultured for 2–3 weeks at 37 °C. When mixed glial cultures were completely confluent, microglia were separated from astrocytes by shaking culture flasks at regular cell-orbital shaker at 37 °C for 1 h. Microglia floating in the medium were collected without disrupting the astrocyte layer on the flask surface and cultured for experimental analysis. Flasks with remaining astrocytes were refilled with grow medium and continued shaking overnight. The next day astrocytes were seeded in plates for further experimental analysis.

### 3.6. Crypt Isolation, Culturing and Live Imaging

Crypt isolation was performed as described previously [23]. Briefly, Intestines were harvested and washed with 1× PBS. Villi were scraped and the remaining tissue were cut into 2 cm pieces and incubated in 2 mM EDTA/PBS for 30 min at 4 °C. Crypts were then collected by shaking and were cultured in Matrigel (BD Bioscience; Heidelberg, Germany; #356231) overlaid with medium containing 50 ng/mL EGF (Life technologies; Darmstadt, Germany; PMG8043), 100 ng/mL mNoggin (Peprotech, #250-38, Cranbury, NJ, USA), 1 μg/mL mR-spondin1 (R&D systems, #2474-RS-050, Minneapolis, MN, USA) (ENR) in the presence of 10 μM Rock-inhibitor (Sigma, Y0503, Setagaya City, Tokyo). Crypts were plated in 8-well Ibidi treat^®^ dishes and imaging was performed on a Leica DMI 6000 confocal microscope equipped with an incubation system and image analysis was completed using Leica LAS AF software (Frankfurt, Germany).

### 3.7. Pancreatic Islet and Exocrine Tissue Isolation and FACS Sorting

To isolate islets and exocrine tissues, we applied adult pancreas digestion as described previously [28,29]. Briefly, we perfused the pancreas by injecting collagenase P (Sigma-Aldrich, Schnelldorf, Germany) dissolved in Hanks Balanced Salt Solution (HBSS) with Ca^2+^/Mg^2+^ into the bile duct. After applying the samples to a gradient solution (5 mL 10% RPM + 3 mL 40% Optiprep/ per sample), we harvested a mixture of islets and exocrine tissues in RPMI medium 1640 supplemented with 11 mM glucose, 10% (*v*/*v*) heat inactivated FBS, 1% (*v*/*v*) penicillin and streptomycin. Samples were then incubated in TriplE for 10 min at 37 °C and resuspended in FACS buffer (PBS with FCS 10%) and filtered through a 35 mm cell strainer that resulted in disaggregation into single cells. Cell sorting and isolation was performed using an Aria III (BD Biosciences, Heidelberg, Germany).

### 3.8. RNA Isolation, RT-PCR and qPCR

For RNA isolation, we washed and homogenized the samples in Qiazol lysis reagent (Qiagen, #79306, Hilden, Germany) or directly sorted them into Qiazol. RNA was extracted using the miRNeasy Micro kit (Qiagen, #217084). We then checked the RNA integrity using Nanodrop (NanoDrop™ 2000/2000c Spektralphotometer) and amplified the cDNA with SuperScript^®^ VILO™ cDNA Synthesis Kit (Thermo Scientific; Darmstadt, Germany; SuperScript IV VILO Master Mix). RT-PCR was performed using primers Syt13 (Mm00600526_m1), GFP, enhanced N+ (Mr04329676_mr), Chra (Mm00514341_m1) and Amy2a3 (Mm02342486_mh). TaqMan qRT-PCR was performed under standard conditions using ViiA7 (Applied Biosystems; Thermo Scientific; Darmstadt, Germany) and TaqMan Fast Advanced Master Mix (Applied Biosystems, #4444557, Waltham, MA, USA). Samples were normalized to housekeeping gene glyceraldehyde 3-phosphate dehydrogenase (Gapdh). We used the following TaqMan probes (Applied Biosystems; Thermo Scientific; Darmstadt, Germany): Chga, Mm00514341_m1, Gapdh, Mm99999915_g1; Syt13, Mm00600526_m1, GFP, Mr04329676_mr, Amy2a3, Mm02342486_mh.

### 3.9. ScRNA-Seq Data Sources and Analysis

Processed single-cell RNA-seq data were obtained from GSE152325 and analyzed using scanpy (v.1.7.1) and anndata (v.0.7.5) in Python v.3.8.5 (Wilmington, DE, USA). Normalized expression values were plotted to indicate Syt13 expression.

## Data Availability

Not applicable.

## Supplementary Materials

The following are available online at https://www.mdpi.com/article/10.3390/ijms222212526/s1, Figure S1: Negative control stainings, Table S1: Primer and gRNA sequences.
