# Supplementary material for: Synaptotagmin-13 Is a Neuroendocrine Marker in Brain, Intestine and Pancreas
Source: Int J Mol Sci. 2021 Nov 20;22(22):12526. doi: 10.3390/ijms222212526 (PMC8620464; doi:10.3390/ijms222212526)
Supplement: Supplementary file 1 [file ijms-22-12526-s001.zip › ijms-1477786-supplementary.pdf]

**Supplementary Information**

# Synaptotagmin-13 is a neuroendocrine marker in brain, intestine and pancreas

Marta Tarquis-Medina[1,2,3], Katharina Scheibner[1,2], Ismael Gonzalez Garcia[2,4], Aimée Bastidas-Ponce[1,2], Michael Sterr[1,2], Jessica Jaki[1,2], Silvia Schirge[1,2], Cristina García-Cáceres[2,4,5], Heiko Lickert[1,2,3*], Mostafa Bakhti[1,2*]

**Supplementary Table S1.** Primer and gRNA sequences

| ID | Sequence 5´- 3´ |
|---|---|
| EP38 | CAAGATCCGCCACAACATCG |
| EP420 | ATTGCATCGCATTGTCTGAGTAG |
| EP1240 | ATTCAACGGCACAGTCAA |
| EP1241 | TGGATGCAGGGATGATGT |
| EP 1640 | CCTACCCGGTAGAATTTCGACGAC |
| EP1774 | CGAATTCCGGCTCACAAGTATC |
| EP1775 | ACCAACTGCACCTGTAGTC |
| EP1771 | GGGTATAGGCATGTGGACAG |
| EP1772 | GGCTTGACTGGACAATGG |
| EP1773 | GCGGACTTGAAGAAGTCG |
| gRNA#1 | ACATGGCGATCTGCCGGCGTGGG |
| gRNA#10 | TGTAGTCGGCCACCTGCCACCGG |

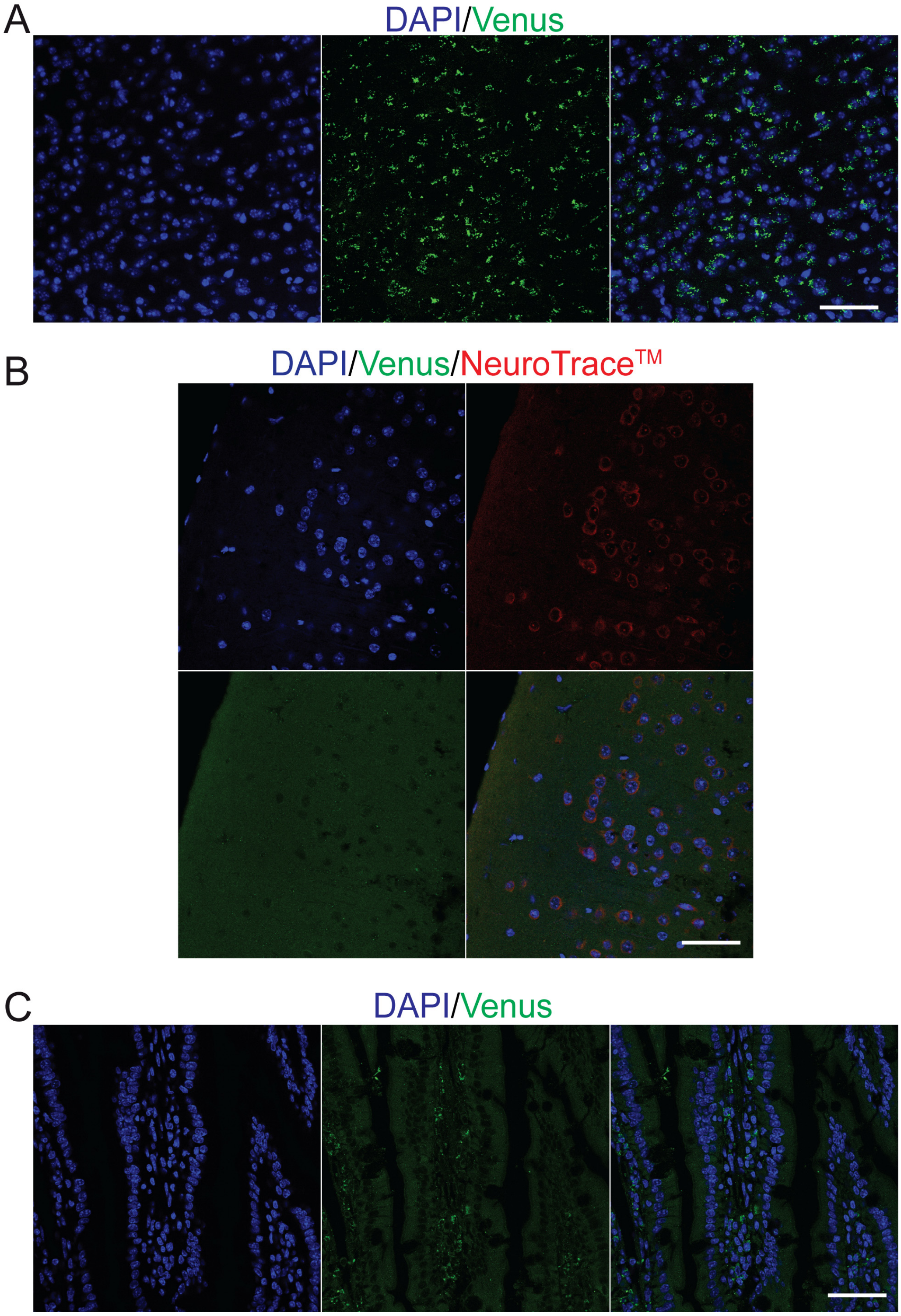


**Supplementary Figure S1. Negative control stainings.** (A) Maximum intensity projection image of cortex area from *Syt13*$^{VF/VF}$ mouse brain. Venus signal (green) is detectable even in the absence of Venus antibody. (B) Brain sections from WT animals stained with antibody against Venus (green) and NeuroTrace™ (red). No overlapping signals is observed between two channels. (C) Staining of duodenal section from WT animals shows a certain degree of unspecific background signal for Venus antibody. Scale bars 50 μm.
